# Identification of Mutant Versions of the Spt16 Histone Chaperone That Are Defective for Transcription-Coupled Nucleosome Occupancy in *Saccharomyces cerevisiae*

**DOI:** 10.1534/g3.112.002451

**Published:** 2012-05-01

**Authors:** Sarah J. Hainer, Brittany A. Charsar, Shayna B. Cohen, Joseph A. Martens

**Affiliations:** Department of Biological Sciences, University of Pittsburgh, Pittsburgh, Pennsylvania 15260

**Keywords:** FACT, Spt16, transcription, chromatin, intergenic DNA

## Abstract

The highly conserved FACT (Facilitates Chromatin Transactions) complex performs essential functions in eukaryotic cells through the reorganization of nucleosomes. During transcription, FACT reorganizes nucleosomes to allow passage of RNA Polymerase II and then assists in restoring these nucleosomes after RNA Polymerase II has passed. We have previously shown, consistent with this function, that Spt16 facilitates repression of the *Saccharomyces cerevisiae SER3* gene by maintaining nucleosome occupancy over the promoter of this gene as a consequence of intergenic transcription of *SRG1* noncoding DNA. In this study, we report the results of a genetic screen to identify mutations in *SPT16* that derepress *SER3*. Twenty-five *spt16* mutant alleles were found to derepress *SER3* without causing significant reductions in either *SRG1* RNA levels or Spt16 protein levels. Additional phenotypic assays indicate that these mutants have general transcription defects related to altered chromatin structure. Our analyses of a subset of these *spt16* mutants reveal defects in *SRG1* transcription-coupled nucleosome occupancy over the *SER3* promoter. We provide evidence that these mutants broadly impair transcription-coupled nucleosome occupancy at highly transcribed genes but not at lowly transcribed genes. Finally, we show that one consequence shared by these mutations is the reduced binding of mutant Spt16 proteins across *SRG1* and other highly transcribed genes. Taken together, our results highlight an important role for Spt16 in orchestrating transcription-coupled nucleosome assembly at highly transcribed regions of the genome, possibly by facilitating the association of Spt16 during this process.

In eukaryotes, genomic DNA is packaged with proteins to form chromatin, a repeating array of nucleosomes that contain 147 bp of DNA wrapped around an octamer of histone proteins (Luger *et al.* 1997). In general, this stable association of DNA and histone proteins poses a significant obstacle to many cellular processes, including transcription, DNA replication, and DNA repair, that rely on proteins being able to interact with DNA [reviewed in Bai and Morozov (2010), Duina (2011), Li *et al.* (2007), and Luger (2006)]. Not surprisingly, eukaryotes express a large group of factors with a range of activities that contribute to the reorganization of chromatin to facilitate these processes. These include chromatin remodelers that use the energy of ATP hydrolysis to reposition or remove histones, posttranslational histone modifiers that covalently add chemical moieties (acetyl, methyl, phosphate, ubiquitin groups) to histone residues that alter their function, and histone chaperones that interact with histones to contribute to the disassembly and reassembly of nucleosomes [reviewed in Cairns (2009), Narlikar *et al.* (2002), and Smith and Shilatifard (2010)].

The highly conserved heterodimer FACT (Facilitates Chromatin Transactions) is a prominent member of the histone chaperone family with reported functions in multiple nuclear processes including DNA replication, DNA repair, transcription initiation, and transcription elongation [reviewed in Duina (2011), Formosa (2008, 2011), and Winkler and Luger (2011)]. Its role in transcription elongation has been particularly well supported by both genetic and biochemical experiments involving yeast and mammalian systems (Formosa 2011). These include the sensitivity of yeast FACT mutants to the transcription elongation inhibitor 6-azauracil, the genetic interaction of these mutants with other known elongation factors, the colocalization of FACT with RNA Polymerase II (Pol II) across transcribed regions of eukaryotic genomes, the physical association of FACT with other transcription elongation factors, and the requirement of human FACT to allow RNA Pol II to transcribe a nucleosomal DNA template *in vitro* (Belotserkovskaya *et al.* 2003; Formosa *et al.* 2001, 2002; Krogan *et al.* 2002; Orphanides *et al.* 1998; Simic *et al.* 2003; Squazzo *et al.* 2002). Although the precise molecular functions of FACT in transcription elongation remain under investigation, several studies have strongly implicated FACT in facilitating the nucleosome dynamics that occur during transcription elongation. These studies suggest that FACT associates with a nucleosome in front of RNA Pol II resulting in the reorganization of histones that eventually lead to the displacement of H2A-H2B dimers and the passage of RNA Pol II (Belotserkovskaya *et al.* 2003; McCullough *et al.* 2011; Orphanides *et al.* 1998). Once RNA Pol II has passed, FACT is also required to assist in the reassembly of nucleosomes to protect recently transcribed DNA from spurious transcription from cryptic intragenic promoters (Belotserkovskaya *et al.* 2003; Formosa *et al.* 2002; Jamai *et al.* 2009; Orphanides *et al.* 1999; Schwabish and Struhl 2004; Stuwe *et al.* 2008; VanDemark *et al.* 2008).

Although a role for FACT in facilitating transcription-dependent nucleosome dynamics has been well documented, less is known concerning the precise contribution of the individual FACT subunits. Yeast FACT is composed of two proteins, Spt16 and Pob3, that are essential for viability and can bind nucleosomes *in vitro* when aided by a third protein, the HMG box-containing protein Nhp6 (Formosa *et al.* 2001; Wittmeyer and Formosa 1997). Pob3 consists of three separate domains defined by limited proteolysis: an N-terminal (NT/D) domain that is thought to be involved in dimerization with Spt16, a middle (M) domain that contains a double pleckstrin homology motif, and an acidic C-terminal (C) domain (Liu *et al.* 2010; VanDemark *et al.* 2006). The pleckstrin homology motif has been implicated in assisting the interactions between FACT and RPA, an essential protein involved in DNA replication and repair (VanDemark *et al.* 2006). Spt16 has been characterized as having four distinct domains, referred to as the N-terminal (NTD), dimerization (D), middle (M), and C-terminal (C) domains (VanDemark *et al.* 2006, 2008). Structures of Spt16-NTD, the one domain that is dispensable for viability, from both *S. cerevisiae* and *S. pombe* have recently been solved by X-ray crystallography, revealing a motif that is structurally similar to bacterial aminopeptidases (Stuwe *et al.* 2008; VanDemark *et al.* 2008). Although interactions between the Spt16-NTD and histones H2A, H3, and H4 have been reported, the fact that this domain is expendable for Spt16 functions *in vivo* suggests that there are likely to be other regions of Spt16 that functionally and physically interact with histones (O’Donnell *et al.* 2004; VanDemark *et al.* 2008). The Spt16-D domain is thought to interface with the NT/D domain of Pob3 to form the FACT dimer (VanDemark *et al.* 2006). Although molecular functions of the Spt16-M domain are not known, mutations altering residues within this domain have resulted in phenotypes indicative of transcription initiation and elongation defects, defects in replication, and defects in cell wall integrity, indicating the functional significance of this domain (Myers *et al.* 2011; O’Donnell *et al.* 2009; Stevens *et al.* 2011). Spt16-C is an acidic domain that is essential for viability whose most 3′ end has recently been shown to functionally interact with histone H3 (Belotserkovskaya *et al.* 2003; Evans *et al.* 1998). Recent *in vitro* analysis of the human Spt16-C domain has implicated this domain in the active displacement of nucleosomal DNA during nucleosome reorganization (Winkler *et al.* 2011).

We have recently provided evidence that FACT contributes to a new mechanism of gene regulation operating at the *S. cerevisiae SER3* gene based on its ability to promote transcription-coupled nucleosome dynamics (Hainer *et al.* 2011; Martens *et al.* 2004). In the presence of serine, transcription of intergenic *SRG1* DNA initiates 5′ of the adjacent *SER3* gene, which encodes an enzyme for serine biosynthesis (Martens *et al.* 2004, 2005). As a consequence of *SRG1* transcription across the *SER3* promoter, FACT assists in the assembly and maintenance of nucleosomes over this region that is otherwise depleted of nucleosomes (Hainer *et al.* 2011). The presence of these nucleosomes at the *SER3* promoter inhibits the binding of transcription factors required to induce *SER3* transcription. In this report, we present the results of an unbiased genetic screen to identify mutations of *SPT16* that derepress *SER3* transcription. Our analyses of these mutants indicate that the integrity of both the Spt16-D and Spt16-M domains not only are required for *SRG1* transcription-dependent nucleosome assembly and *SER3* repression but also are more broadly required for transcription-coupled nucleosome occupancy at highly transcribed genes. We provide evidence suggesting a possible role for the Spt16-D and Spt16-M domains in promoting the association of FACT to genes being actively transcribed.

## Materials and Methods

### Strains and media

All *S. cerevisiae* strains used in this study (supporting information, Table S1) are isogenic to a *GAL2^+^* derivative of S288c (Winston *et al.* 1995). All strains were constructed by transformation or by genetic crosses (Ausubel *et al.* 1991). YJ920 and YADP50 have been previously described (Hainer *et al.* 2011; Myers *et al.* 2011). Strains YJ1089-YJ1092 were derived from YJ920. The *spt16*Δ::*KanMX* and *lys2-128*δ alleles have been previously described (Clark-Adams *et al.* 1988; Myers *et al.* 2011). The *lyp1*Δ::*SER3*pr*-HIS3* allele was generated by replacing the *URA3* open reading frame (ORF) in *lyp1*Δ::*SER3*pr*-URA3* (Hainer and Martens 2011) with a PCR product containing the *HIS3* open reading frame that was amplified from pRS403 (Sikorski and Hieter 1989). pAO01 and p*SPT16-URA3* are centromeric plasmids marked with *LEU2* and *URA3*, respectively, that contain wild-type *SPT16* (kindly provided by A. Duina) (Myers *et al.* 2011). Derivatives of pAO01 containing *spt16-G132D* and *spt16-T828I/P859S* alleles were generated by standard cloning methods and verified by sequencing. All other *spt16* mutants characterized in this study are expressed from plasmids derived from pAO01. Yeast extract-peptone-dextrose (YPD), synthetic complete (SC), omission (SC−), 5-fluoroorotic acid (5-FOA), and galactose media have been previously described (Rose 1991). YPD was supplemented with 5 μg/mL cyclohexamide (CHX) or 200 mM hydroxyurea (HU) as indicated. 3-amino-1,2,4-triazole (3-AT; Sigma) was added to SC medium lacking leucine and histidine at the indicated concentrations.

### Screen for *spt16* mutants that derepress *SER3*

Using a previously described strategy (Myers *et al.* 2011), two regions of *SPT16*, from +764 to +2044 (region B) and from +1430 to +3521 (region C), were amplified from pAO01 plasmid (gift from A. Duina) using GoTaq polymerase (Invitrogen) and standard PCR conditions. Amplified DNA was cotransformed into YJ1089 with pAO01 plasmid that had been digested with either *Eag*1 and *Sna*B1 (region B) or *Sna*B1 and *Xba*I (region C). Transformants containing gap-repaired plasmids were selected on SC medium lacking leucine and then replica-plated onto medium containing 5-FOA to select for cells that lost the *URA3*-marked plasmid carrying a wild-type copy of *SPT16* (p*SPT16-URA3*). The resulting colonies were then replica-plated to SC medium lacking histidine and leucine that was supplemented with 5 mM 3-AT. Candidate plasmids were recovered from strains resistant to 5 mM 3-AT, retransformed into YJ1089, and retested for their ability to confer 3-AT resistance. The region of *SPT16* that was subjected to PCR mutagenesis was first subcloned into a new copy of pAO01 before retransformation. For each plasmid that retested for 3-AT resistance, both strands of the entire *SPT16* gene were sequenced and compared with the wild-type gene.

### Northern analysis

Cells were grown to approximately 2 × 10^7^ cells/ml in YPD at 30°. Total RNA isolation and Northern analysis was performed as previously described (Collart and Oliviero 2001). Radiolabeled DNA probes to *SRG1* (−454 to −123 relative to *SER3* ATG), *SER3* (+111 to +1342), and *SCR1* (−163 to +284) were generated by random-primed labeling of PCR fragments amplified from genomic DNA. RNA levels were quantified using a PhosphorImager (FLA-5000) and ImageJ software.

### Western analysis

Whole cell extracts (WCE) were prepared from cells grown in YPD at 30° to approximately 3 × 10^7^ cells/ml using trichloroacetic acid as previously described (Cox *et al.* 1997; Zheng *et al.* 2010). Equal amounts of WCE were separated by 10% acrylamide SDS-PAGE, transferred to Protean nitrocellulose (Whatman), and assayed by immunoblotting. The antibodies used to detect Spt16, Pob3, and G6PDH were as follows: anti-Spt16 (1:500; gift from T. Formosa), anti-Pob3 (1:2000; gift from T. Formosa), anti-G6PDH (1:50,000; Sigma). After incubation with HRP-conjugated IgG secondary antibody (1:5000; GE Healthcare), the immunoreactive proteins were visualized by enhanced chemiluminescence detection (PerkinElmer) using a Kodak image station 440*CF*. Spt16 and Pob3 protein levels were calculated by measuring their signal intensities in these Western blots using Kodak ID 3.6 software and normalizing these values to those obtained for the G6PDH control.

### Dilution growth assays

Cells were grown at 30° overnight to saturation then washed twice with water. Starting at 1 × 10^8^ cells/ml, cultures were serially diluted 10-fold. Three microliters of each dilution was spotted onto indicated media and incubated at 30° for the indicated number of days.

### Nucleosome scanning assay

Cells were grown at 30° to approximately 2 × 10^7^ cells/ml in YPD and subjected to a nucleosome-scanning assay, as previously described (Hainer *et al.* 2011). For each of the 38 *SER3* primer pairs, the amount of template protected from digestion by micrococcal nuclease (MNase) was calculated as a ratio between MNase-digested and MNase-undigested samples and then normalized to the amount of MNase-protected control template (*GAL1* NB) that is located within a well-positioned nucleosome in the *GAL1* promoter (Brickner *et al.* 2007; Floer *et al.* 2010).

### Chromatin immunoprecipitation (ChIP)

Cells were grown in YPD at 30° to approximately 2 × 10^7^ cells/ml. Chromatin was prepared as previously described (Shirra *et al.* 2005). Histone H3, Spt16, or Rpb3 were immunoprecipitated by incubating sonicated chromatin overnight at 4° with 1 μl anti-histone H3 antisera [previously described in Tomson *et al.* (2011)], 1 μl anti-Spt16 antisera (kindly provided by T. Formosa), or 2.5 μl anti-Rpb3 antisera (W0012; Neoclone) followed by the addition of IgG-Sepharose beads (GE Healthcare) for 2 hr at 4°. Dilutions of input DNA and immunoprecipitated DNA were analyzed by qPCR reactions. Primer sets that amplify the following regions were used for qPCR: *SER3*-41 (−921 to −828, relative to +1 ATG of *SER3*); *SER3*-25 (−338 to −289, relative to +1 ATG of *SER3*); *SER3*-22 (−300 to −200, relative to +1 ATG of *SER3*); *SER3*-7 (+195 to +295); *PYK1* (5′: +62 to +164, 3′: +1173 to +1279); *PMA1* (5′: +691 to +794, 3′: +1689 to +1791); *ADH1* (+845 to +943); *CYC1* (+122 to +217); *TUB2* (5′: +105 to +202, 3′: +1083 to +1189); and *GAL1* (5′: +79 to +175, 3′: +1366 to + 1487). Histone H3, Spt16, and Rpb3 ChIP signals for each gene were normalized to a No ORF control template, which is located within a region of chromosome V that lacks open reading frames (Komarnitsky *et al.* 2000).

### Quantitative PCR (qPCR)

All qPCR data for the nucleosome scanning and ChIP assays were obtained by using an ABI StepOne Plus Real-time system using SYBR green reagents (Fermentas) and the indicated primers (Hainer *et al.* 2011). Calculations were performed using Pfaffl methodology (Pfaffl 2001).

## Results

### Identification of *spt16* mutants that derepress *SER3*

**Figure 1:**
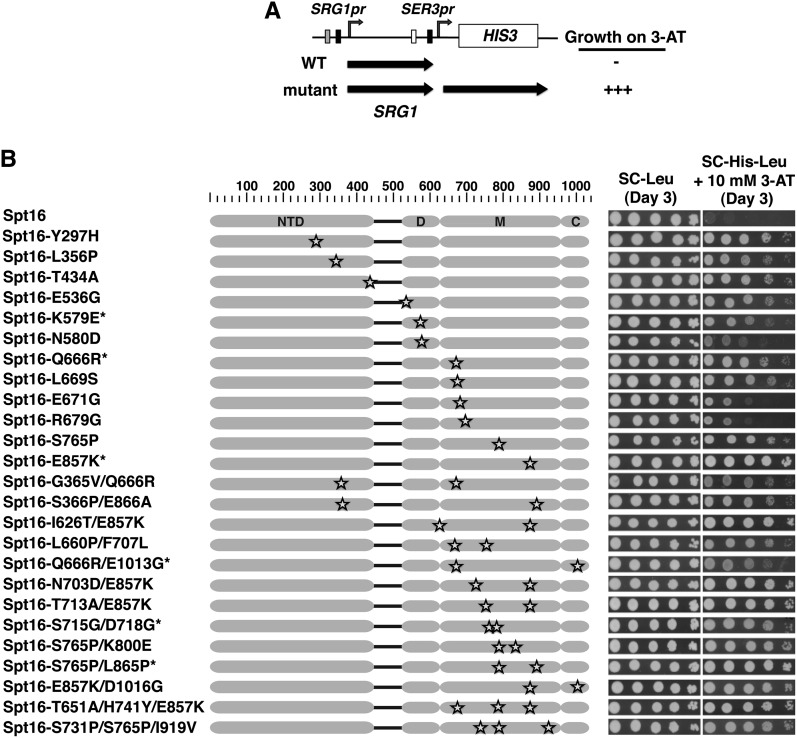
Identification of *spt16* mutants that derepress an ectopically expressed *SER3*pr*-HIS3* reporter gene. (A) Diagram of *SER3*pr*-HIS3* reporter. The *LYP1* ORF was replaced by *SER3* intergenic sequence from −713 to −1, including *SRG1* and its promoter, fused to the *HIS3* ORF. Block arrows beneath the diagram indicate the expected *SRG1* and *SER3-HIS3* transcripts in wild-type and mutant strains grown in serine-rich media (YPD). The expected growth of these strains on SC-His-Leu plates containing 3-AT is indicated on the right. (B) Growth assays indicating that newly isolated *spt16* mutants derepress *SER3*pr*-HIS3* reporter. *spt16*Δ cells (YJ1089) containing *LEU2*-marked plasmids expressing either wild-type or mutant Spt16 protein, as indicated, were grown to saturation in YPD, diluted to 10^8^ and then spotted in a 10-fold serial dilution series on SC-His-Leu (control) and SC-His-Leu + 10 mM 3-AT plates. Plates were incubated at 30° for three days. Results were obtained for two independent growth assays in which each plate contained control strains and five to six mutants. Shown are representative dilutions for the control strains and each *spt16* mutant strain. Each Spt16 mutant protein is named to describe the location and nature of the amino acid substitution. The locations of the amino acid substitutions in each of these mutants are also indicated (marked by stars) in diagrams of Spt16; gray ovals indicate the N-terminal (NTD), dimerization (D), middle (M) domains, and the C-terminal acidic tail region (C). Note that the five mutants marked by an asterisk each have an additional silent mutation.

We recently described a new mechanism of gene regulation in *Saccharomyces cerevisiae* whereby transcription of *SRG1* ncDNA assembles nucleosomes over the promoter of the adjacent *SER3* gene to maintain *SER3* repression (Hainer *et al.* 2011). Furthermore, we provided evidence that the histone chaperones, Spt6 and Spt16, are required to maintain this nucleosome occupancy, and repress *SER3*, likely through their ability to disassemble and reassemble nucleosomes during active transcription (Belotserkovskaya *et al.* 2003; Hainer *et al.* 2011). To investigate the role of Spt16 in this mechanism, we performed an unbiased genetic screen to identify novel mutations in *SPT16* that derepress *SER3* during *SRG1* transcription. A PCR-based strategy that has been previously described [see *Materials and Methods*; Myers *et al.* (2011)] was used to target mutagenesis of the 3′ half of *SPT16* that excludes most of the N-terminal domain (NTD), which is dispensable for *SER3* repression (S. J. Hainer, unpublished data). These PCR fragments were cotransformed with a gapped *LEU2*-marked plasmid that contained homology to the PCR fragments into an *spt16Δ his3Δ* strain containing an integrated *SER3*pr*-HIS3* reporter (Figure 1A) and expressing a wild-type copy of *SPT16* from a *URA3*-marked plasmid (YJ1089). Following gap-repair and loss of the *URA3*-marked plasmid expressing *SPT16*, we screened for *spt16* mutants that derepress the *SER3*pr*-HIS3* reporter by their ability to confer growth in the presence of 3-AT, a competitive inhibitor of the *HIS3* gene product (Figure 1).

With this screen, we initially identified 522 mutants that permit growth on medium containing 5 mM 3-AT. Forty *SPT16*-containing plasmids were then recovered from strains that conferred resistance up to 40 mM 3-AT to enrich for mutations that most strongly derepress *SER3*. The remaining 482 plasmids have not yet been examined. After retesting for their ability to derepress the *SER3*pr*-HIS3* reporter, the entire *SPT16* gene contained on each of these plasmids was sequenced. Sequencing of the 38 plasmids that successfully retested identified 25 unique *spt16* mutants harboring nucleotide changes that result in either single (12), double (11), or triple (2) amino acid substitutions. For the 12 single amino acid substitution mutants, the location of the altered amino acids varies: 3 are located at the very 3′ end of the Spt16-NTD, 3 are found in Spt16-D, and the remaining 6 are found in Spt16-M, including 3 residues that are within 13 amino acids of each other (Figure 1B). Interestingly, 9 of the 13 double or triple mutants contain 1 of the single amino acid substitutions, indicating that the effect on the *SER3* reporter from these mutation combinations is likely through the isolated single substitution. Interestingly, only one of these mutations, *spt16-E857K*, has been previously reported (O’Donnell *et al.* 2009; Stevens *et al.* 2011). In these studies, *spt16-E857K* was isolated as a dominant suppressor of a transcription defect caused by the insertion of a δ element 5′ of the *LYS2* and *HIS4* genes (Spt^−^ phenotype) and was found to genetically interact with mutations in other transcription elongation factors.

### Phenotypic analysis of the *spt16* mutants

**Figure 2:**
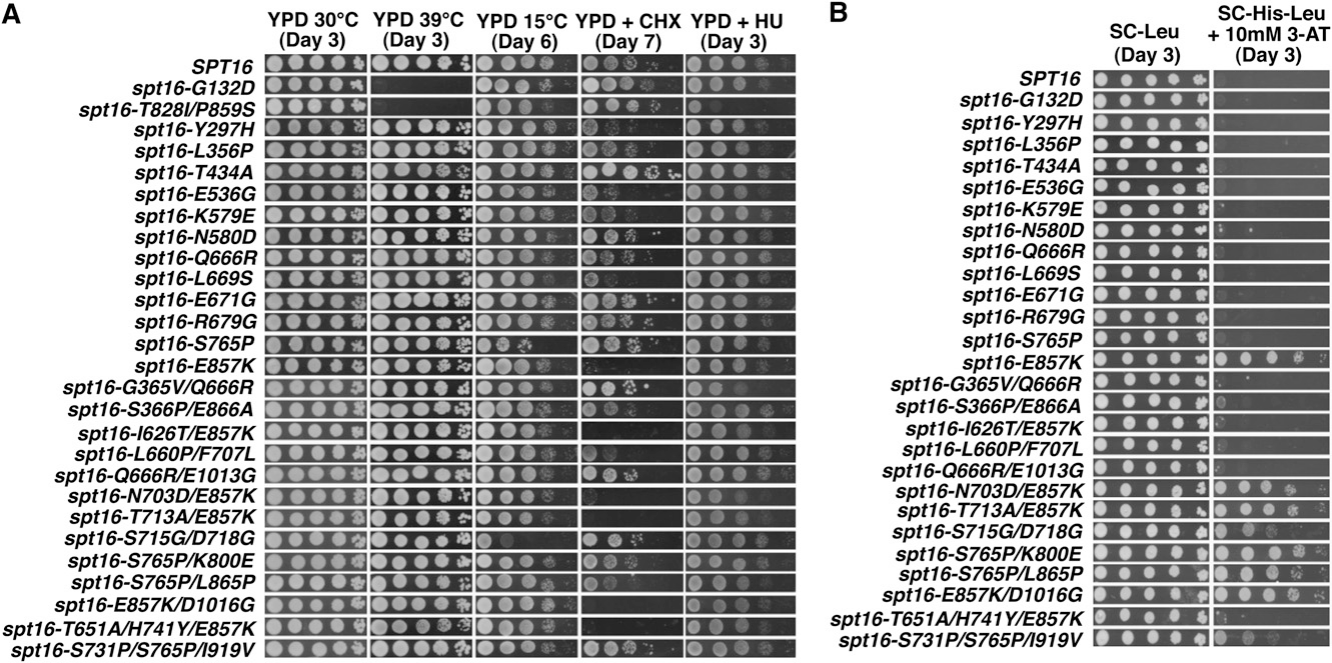
Phenotypic characterization of newly isolated *spt16* mutants. (A) Temperature, cyclohexamide (CHX), and hydroxyurea (HU) sensitivity of *spt16* mutants. *spt16*Δ cells (YJ1091) expressing either wild-type or the indicated mutant alleles of *SPT16* from a *LEU2*-marked plasmid were grown to saturation in YPD at 30°, diluted to 10^8^, spotted in 10-fold serial dilutions, and incubated for the indicated number of days on solid media. Cells spotted on YPD were incubated at 30°, 39°, or 15°, and cells spotted on YPD+CHX or YPD+HU were grown at 30°. Results are a representative of two biological replicates. (B) Dominance test. Cells expressing wild-type *SPT16* from its genomic location and the *SER3*pr-*HIS3* reporter (YJ1090) were transformed with plasmids containing either wild-type or the indicated mutant *SPT16* alleles. Transformants were grown to saturation in YPD at 30° and spotted on SC-His-Leu (control) and SC-His-Leu + 10 mM solid medium, which were then incubated at 30° for three days. Results are representative of two biological replicates.

To further characterize these mutants, we tested the strains for temperature sensitivities and growth defects on YPD medium supplemented with cyclohexamide (CHX), hydroxyurea (HU), mycophenolic acid (MPA), and caffeine. Surprisingly, we found that not one of the *spt16* mutants that we isolated confers a growth defect at elevated temperatures (39°) or in the presence of HU (Figure 2A), phenotypes that have been previously described for other *spt16* alleles, including *spt16-G132D* (Figure 2A, row 2) and *spt16-T828I/P859S* (Figure 2A, row 3) (Formosa *et al.* 2001). Interestingly, one mutant, *spt16-S715G/D718G*, confers cold sensitivity at 15°, and a number of the mutants cause varying sensitivities to CHX (Figure 2A). No detectable growth defects were observed when strains expressing any one of the isolated *spt16* mutants were exposed to MPA or caffeine (S. B. Cohen, unpublished data).

We also tested whether the *spt16* mutants that we isolated are dominant for repression of the *SER3*pr*-HIS3* reporter. YJ1090 cells containing wild-type *SPT16* at its genomic location, *SER3*pr*-HIS3*, and a plasmid expressing either wild-type or mutant version of Spt16 were spotted onto media containing 3-AT to test for expression of the *SER3*pr*-HIS3* reporter. One mutant allele, *spt16-E857K*, is dominant for derepression of the *SER3* reporter gene, suggesting that it may be a gain-of-function mutation (Figure 2B). Interestingly, our analysis of the more complex mutants identified amino acid substitutions at *I626T* and one or both of *T651A* and *H471Y* as intragenic suppressors of the dominant effect of this *E857K* substitution. Additionally, our analysis revealed that whereas the mutant *spt16-S765P* allele alone does not confer dominance, it is synthetically dominant for *SER3* derepression with either a *K800E* or *L865P* substitution mutation.

### *spt16* mutants derepress endogenous *SER3*

**Figure 3:**
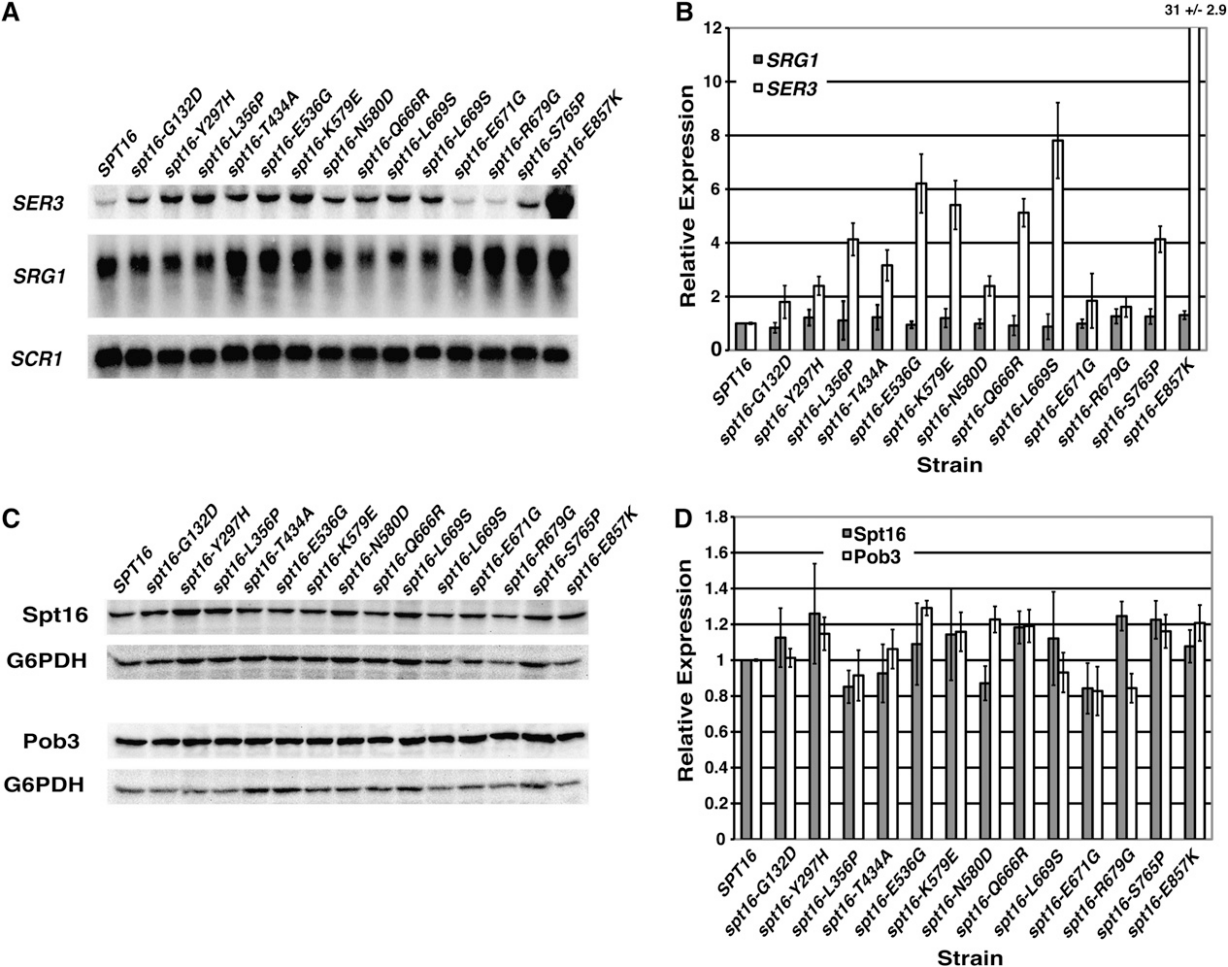
Single amino acid substitutions in Spt16 strongly derepress endogenous *SER3*. (A) Northern blot analysis examining the effect of *spt16* mutants on *SER3*, *SRG1*, and *SCR1* (loading control). Total RNA was isolated from *spt16*Δ cells (YJ1091) carrying plasmid-borne wild-type or mutant *SPT16* alleles that were grown to a density of ∼2 × 10^7^ cells/ml in YPD at 30°. Note that there are two independent strains with the *spt16-L669S* mutation. (B) Quantitation of Northern data. *SRG1* (gray bars) and *SER3* (white bars) RNA levels for the *spt16* mutants are normalized to the level of the *SCR1* loading control and are relative to strains expressing wild-type *SPT16* (arbitrarily set to 1). Each bar indicates the mean RNA level ± SEM from four independent experiments using two transformations each of YJ1091 and YJ1092. (C) Western analysis examining the effect of *spt16* mutant alleles on mutant Spt16 and Pob3 protein levels. Whole cell extracts were prepared from the same set of strains described in panel A grown to ∼3 × 10^7^ cells/ml in YPD at 30° and subjected to Western analysis using anti-Spt6 and anti-Pob3 antibodies (kindly provided by T. Formosa). Blots were reprobed with anti-G6PDH antibody as a loading control. Note that there are two independent *spt16-L669S* mutants. (D) Quantitation of Western data. Spt16 (gray bars) and Pob3 (white bars) protein levels are normalized to the G6PDH loading control and are relative to strains expressing wild-type *SPT16* (arbitrarily set to 1). Each bar indicates the mean protein level ± SEM from three independent experiments using the same set of strains as in panel B.

We next determined the effect of these *spt16* mutants on endogenous *SER3* and *SRG1* RNA levels. We transformed plasmids containing either wild-type *SPT16*, a previously characterized *spt16-G132D* mutant (Malone *et al.* 1991), or one of our newly isolated *spt16* mutants into YJ1091 and YJ1092 strains and performed Northern assays on these strains (Figure 3, A and B). For these and subsequent experiments, we limited our analysis to the 12 *spt16* mutants having single amino acid substitutions. All of the *spt16* mutants tested derepress *SER3* with effects ranging from very strong (30-fold increase for *spt16-E857K*) to milder (2-fold increase for *spt16-Y297H*, *spt16-N580D*, *spt16-E671G*, and *spt16-S765P*) that are similar to what we had previously observed for *spt16-G132D* (Hainer *et al.* 2011). Although we did observe some variability in *SRG1* RNA levels between experiments, average results from four independent experiments indicate that these *spt16* mutants do not significantly alter *SRG1* RNA levels. Consistent with these Northern data, we found equivalent levels of RNA Pol II localized across the *SRG1* transcription unit in strains expressing either wild-type or mutant versions of Spt16 (Figure 6B). Moreover, Western analyses showed that these newly isolated *spt16* mutants do not alter the levels of Spt16 or its interacting partner, Pob3 (Figure 3, C and D). Taken together, these data identify amino acids in Spt16 that are critical for *SER3* repression.

### Effect of *spt16* mutants on nucleosome occupancy over the *SER3* promoter

**Figure 4:**
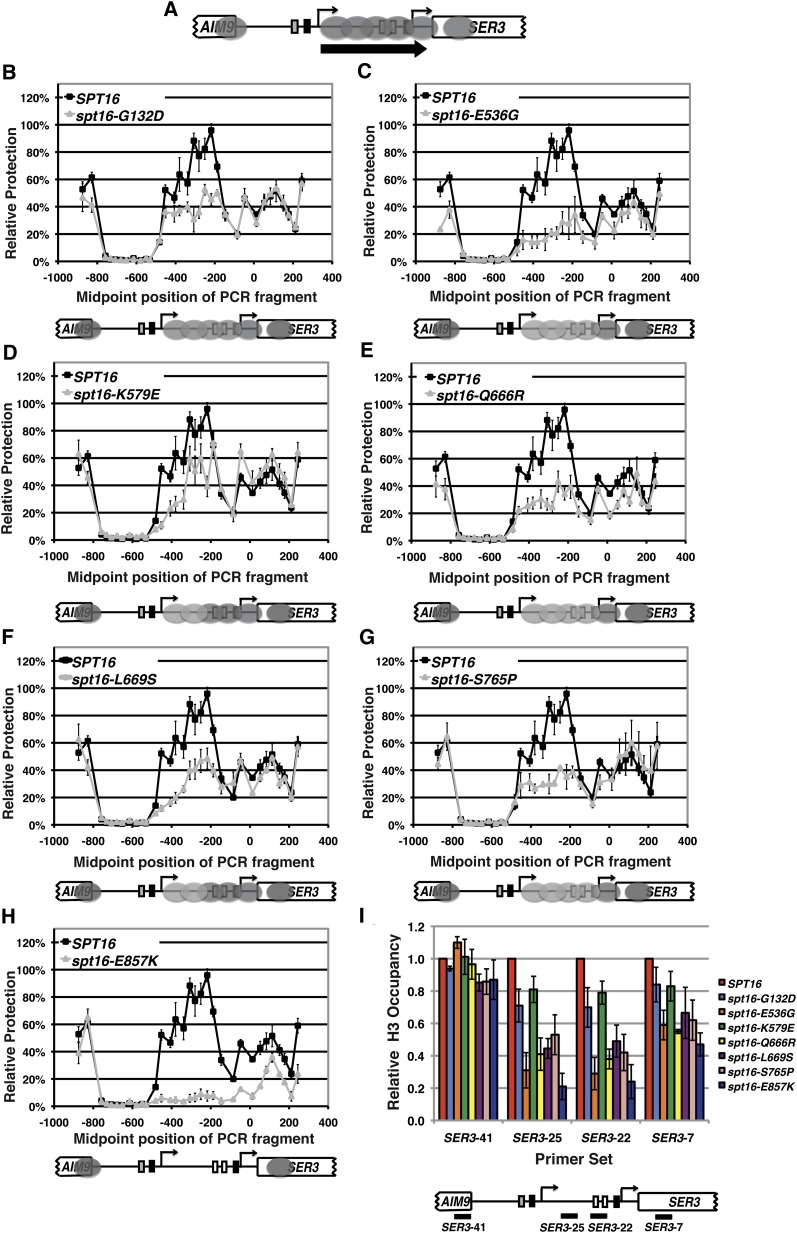
Effect of *spt16* mutants on chromatin structure at *SER3*. (A) Diagram of the *SER3* locus. The gray ovals mark the position of nucleosomes when wild-type cells are grown in *SER3*-repressing conditions (YPD). The block arrow indicates *SRG1* transcription. (B–H) Nucleosome scanning assays were performed on *spt16*Δ cells (YJ1091 and YJ1092) carrying plasmids expressing either wild-type *SPT16* or mutant *spt16* alleles as indicated. Mononucleosome-sized DNA fragments were generated by micrococcal nuclease (MNase) digestion of formaldeyde-treated chromatin that was isolated from cells grown to ∼2 × 10^7^ cells/mL in YPD media at 30°. MNase protection across the *SER3* locus relative to a positioned nucleosome within the *GAL1* promoter was determined by qPCR. For each PCR amplicon, the mean MNase protection ± SEM from three independent experiments is plotted at its midpoint. Shown below each graph is a diagram of the *SER3* locus indicating the positions of nucleosomes (gray ovals) extrapolated from the MNase protection data for each *spt16* mutant. The light-gray ovals are indicative of less dramatic reductions in MNase protections compared with the wild-type control shown in panel A. (I) Histone H3 ChIP was performed on chromatin isolated from the same strains used in panels B–H. The amount of immunoprecipitated DNA was determined by qPCR as a fraction of the input that was then normalized to a control region in chromosome V and made relative to strains expressing wild-type *SPT16* (arbitrarily set to 1). Each bar represents the mean ± SEM of three independent experiments. Below the graph is a schematic of *SER3* with black bars corresponding to the regions amplified by qPCR.

To examine the effect of a subset of the *spt16* mutants on nucleosome occupancy at *SER3*, we performed nucleosome-scanning assays on six of the single amino acid substitutions that most strongly derepress *SER3* (Figure 4). As previously described (Hainer *et al.* 2011), MNase protection across *SER3* was normalized to the protection of a well-studied, nucleosome-bound region of the *GAL1* promoter whose digestion by MNase is unaffected by these *spt16* mutants (S. J. Hainer, unpublished data; see *Materials and Methods* for details). Compared with strains containing wild-type control plasmids, protection from MNase digestion was reduced across the *SRG1*-transcribed region in all the *spt16* mutants examined to degrees approximately equal to or exceeding that of *spt16-G132D* (Figure 4), which we had previously shown to decrease nucleosome occupancy across the *SER3* locus (Hainer *et al.* 2011). MNase protection across the *SER3* promoter region was most dramatically reduced in the *spt16-E857K* mutant (Figure 4H), which is consistent with the strong derepression of *SER3* that is observed in this mutant. The other six mutants that displayed more modest defects in *SER3* repression also had more modest reductions in the MNase protection across the *SER3* promoter. However, we did observe subtle differences in the MNase protection patterns between these mutants. Two of the *spt16* mutants resulted in greater sensitivity to MNase toward the 5′ of *SRG1* relative to the 3′ of *SRG1* (*spt16-K579E* and *spt16-L669S*), compared with the other mutants that had increases in MNase sensitivity that were more evenly distributed across the *SRG1* transcription unit (Figure 4, compare −400 and −200 regions in panels D and F with panels E and G).

To confirm that the changes in MNase protection across the *SRG1* transcription unit caused by these *spt16* mutants reflect changes in nucleosome occupancy, we measured histone occupancy across this region by ChIP. For the most part, histone H3 occupancy across the *SRG1* transcription unit was reduced in the *spt16* mutants to degrees that correlate with the results of our MNase experiments (Figure 4I). Taken together, these data identify Spt16 residues whose integrity is required to maintain *SER3* repression by facilitating *SRG1* transcription–dependent nucleosome occupancy across the *SER3* promoter.

### Effect of *spt16* mutations on phenotypes associated with defects in transcription and chromatin structure

**Figure 5:**
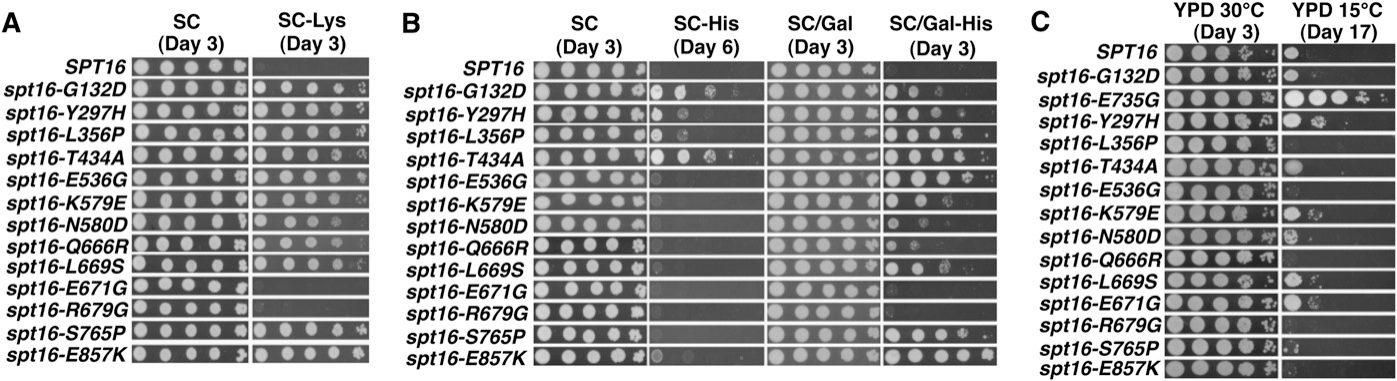
Analysis of *spt16* mutants for phenotypes associated with defects in transcription and chromatin. (A) Assay for Spt^−^ phenotype. *spt16Δ* cells containing the *lys2-128*δ allele and plasmids that express either wild-type or mutant Spt16, as indicated, were grown to saturation in YPD at 30°, diluted to 10^8^, and spotted in 10-fold serial dilutions onto solid synthetic complete medium (SC) and synthetic complete medium lacking lysine (SC-Lys). Plates were incubated at 30° for 3 days. Results are representative of two independent assays using transformations of YJ1091. (B) Assay for cryptic transcription initiation. *spt16Δ* cells containing the *FLO8-HIS3* reporter and plasmids that express either wild-type or mutant Spt16, as indicated, were grown to saturation in YPD at 30°. Serial diluted cells were spotted onto solid synthetic complete medium with or without histidine containing either glucose (SC and SC-His) or galactose (SC/Gal and SC/Gal-His) as a carbon source. Plates were incubated at 30° for either 3 days (SC, SC/Gal, and SC/Gal-His) or 6 days (SC-His). Results are representative of two independent assays using transformants of YJ1092. (C) Assay for suppression of histone H3 L61W mutant. *spt16Δ* cells expressing the H3-L61W mutant as its sole source of histone H3 and plasmids expressing either wild-type or mutant Spt16, as indicated, were grown to saturation in YPD at 30°. Serial diluted cells were spotted onto solid YPD medium as described in panel A and grown at 30° (3 days) or 15° (17 days). Results were generated with strains derived from YADP50 (kindly provided by A. Duina) and are representative of two independent assays.

Having shown a role for at least six of the *spt16* single mutants in regulating chromatin structure at *SER3*, we tested whether all 12 single mutants confer other phenotypes indicative of chromatin-related transcriptional defects. We first determined whether these *spt16* mutants can confer an Spt^−^ phenotype (suppressor of Ty δ element insertion), which is caused by defects in chromatin and aberrant transcription initiation (Clark-Adams *et al.* 1988). *spt16*Δ strains containing the *lys2-128*δ allele were transformed with plasmids containing either wild-type *SPT16* or mutant *spt16* alleles and assayed for their ability to grow on medium lacking lysine (Figure 5A). As a control, we also introduced a plasmid expressing the *spt16-G132D* allele, which has been previously shown to have an Spt^−^ phenotype (Evans *et al.* 1998). Compared with the cells expressing wild-type *SPT16*, most of the *spt16* mutants grew robustly in the absence of lysine, similar to what was observed for the *spt16-G132D* control, indicating that these mutants confer a strong Spt^−^ phenotype (Figure 5A). In contrast, the two *spt16* mutants that most weakly derepress *SER3*, *spt16-E671G* and *spt16-E679G*, had no detectable Spt^−^ phenotype.

Next, we tested whether these *spt16* mutants permit the production of aberrant intragenic transcripts, a phenotype that has been associated with defects in transcription-coupled nucleosome reassembly (Carrozza *et al.* 2005; Kaplan *et al.* 2003). For these experiments, we employed a previously described *GAL*pr-*FLO8-HIS3* reporter gene in which *HIS3* gene expression is dependent on transcription initiation from a cryptic promoter within the *FLO8* coding sequence (Cheung *et al.* 2008). Therefore, cryptic intragenic transcription can be measured by the growth of *his3*Δ cells containing this reporter construct on medium lacking histidine. For this assay, strains expressing plasmid-borne *SPT16* or the indicated *spt16* mutant alleles were monitored for growth on medium lacking histidine. When grown in galactose-containing medium, all but two of the *spt16* mutants allowed cells to grow in the absence of histidine, indicative of robust transcription initiation from the cryptic promoter within the *FLO8* coding sequence (Figure 5B). For the most part, these data correlate well with the Spt^−^ phenotypic data, suggesting that the molecular defects resulting in these two phenotypes are likely related. Interestingly, only those mutations within the N-terminal domain of Spt16 allowed cells to grow in glucose-containing medium lacking histidine, suggesting that these mutants permit cryptic transcription initiation even in the absence of significant levels of transcription across this region.

Finally, we tested the *spt16* mutants for their ability to suppress a cold-sensitive (cs) phenotype of a histone mutant, H3 L61W, a phenotype that has been previously described for a distinct class of mutations located within the Spt16 M-domain (Myers *et al.* 2011). For this assay, *spt16*Δ cells containing the H3 L61W mutant as the sole source of histone H3 (YADP50) and plasmid-borne copies of wild-type or mutant versions of *SPT16* were monitored for growth on YPD at 15° (Figure 5C). When compared with the *spt16-E735G* control (kindly provided by A. Duina), none of our identified *spt16* mutants suppressed the cold sensitivity of H3 L61W. Therefore, the *spt16* mutants we isolated as being defective for *SER3* repression represent a distinct class of mutants from those that suppress the cs phenotype of the H3 L61W mutant and may define functionally distinct regions of the Spt16-D and Spt16-M domains.

### Occupancy of mutant versions of Spt16 is reduced across *SRG1* and the *SER3* promoter region

**Figure 6:**
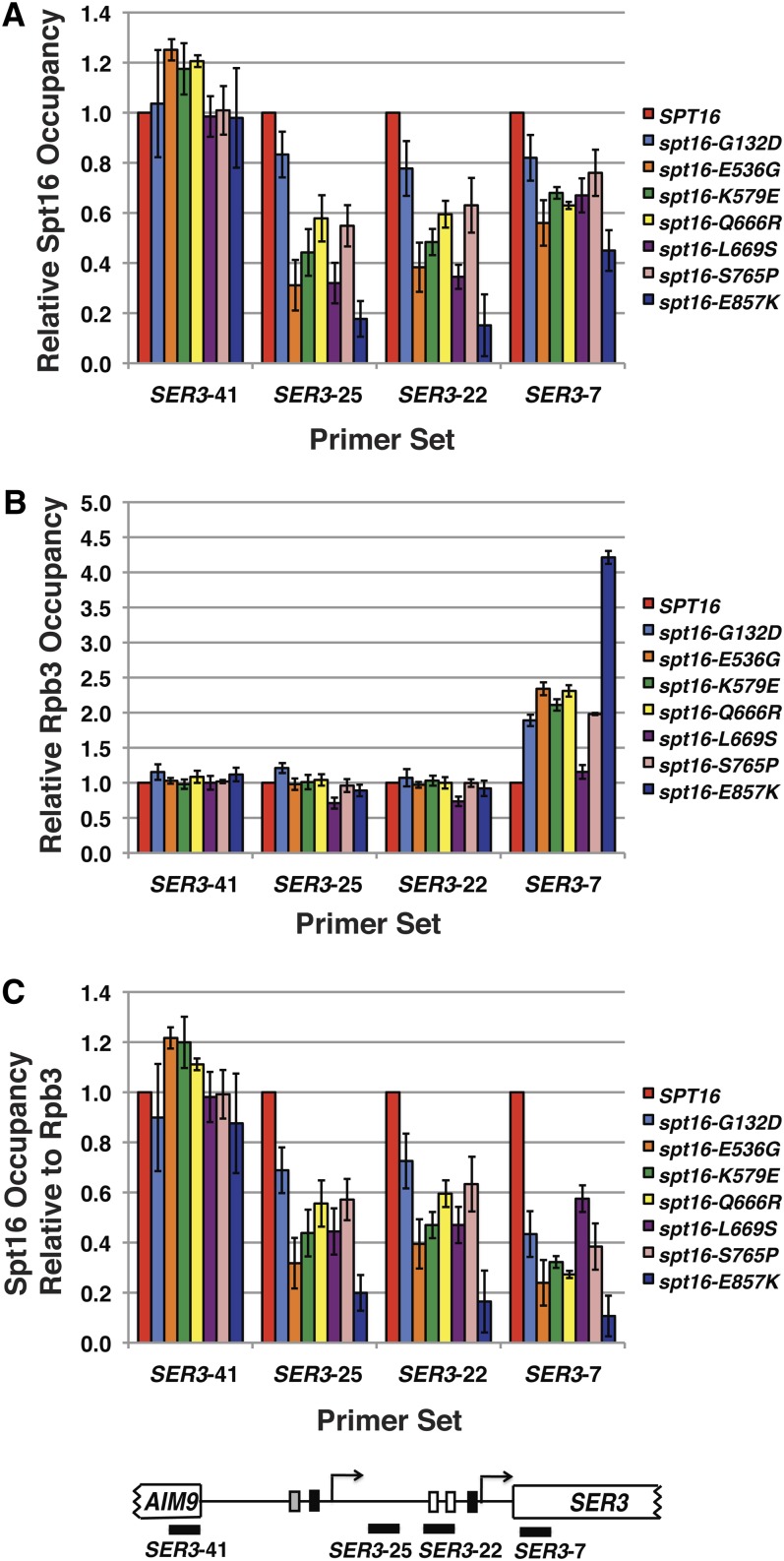
Relative occupancy of Spt16 and RNA Pol II across *SER3* in *spt16* mutants. Spt16 (A) and Rpb3 (B) ChIP experiments were performed on chromatin prepared from *spt16*Δ strains expressing either wild-type or mutant Spt16, as indicated, that were grown in YPD at 30°. The amount of immunoprecipitated DNA at four locations across *SER3* (indicated by black bars in the diagram of *SER3* below the graphs) was determined by qPCR as a fraction of the input material and normalized to a control region in chromosome V. Each bar represents the mean ± SEM of three independent experiments using strains derived from YJ1091 and YJ1092. Occupancy of these factors in the strains expressing wild-type Spt16 was arbitrarily set to 1 at each *SER3* location. (C) Occupancy of Spt16 across *SER3* was recalculated relative to Rpb3 occupancy.

We next considered the possibility that these mutant versions of Spt16 fail to be recruited normally to transcribed regions, which may account for their multiple phenotypes related to defects in transcription-coupled nucleosome occupancy. Therefore, we performed ChIP experiments to assess the binding of selected Spt16 mutant proteins across the *SRG1* transcription unit (Figure 6A). In general, we detected reduced binding of most of the mutant versions of Spt16 that parallels the loss of histone H3 occupancy across this region that we observed in these mutant versions (compare Figure 6A with Figure 4I). The lone exception is the *spt16-K579E* mutant where we detected a stronger decrease in the occupancy of the mutant protein expressed from this allele than expected based on a relatively modest decrease in histone H3 occupancy. Because Spt16 strongly colocalizes with RNA Pol II across transcribed genes, we tested whether the decrease in the occupancy of the mutant versions of Spt16 might be indirect due to a decrease in RNA Pol II occupancy at *SER3*. To this end, we performed ChIP analysis of Rpb3, a subunit of RNA Pol II, over *SRG1* (Figure 6B). Consistent with our Northern analysis (Figure 3), we found that all but one of these *spt16* mutants did not cause a decrease in RNA Pol II occupancy compared with cells expressing wild-type *SPT16*. Interestingly, the *spt16-L669S* mutant did cause a slight but significant decrease (*P* < 0.05) in Rpb3 binding across *SRG1*. However, by normalizing the binding of this mutant version of Spt16 to Rpb3 binding, it is clear that this minor decrease in Rpb3 binding alone cannot account for the reduced binding of this mutant version of Spt16 across *SRG1* (Figure 6C). Taken together, these data indicate that the amino acids defined by these mutants are required to maintain Spt16 colocalization with RNA Pol II across *SRG1*.

### Effect of *spt16* mutants on histone H3, Spt16, and RNA Pol II occupancy at other genes

**Figure 7:**
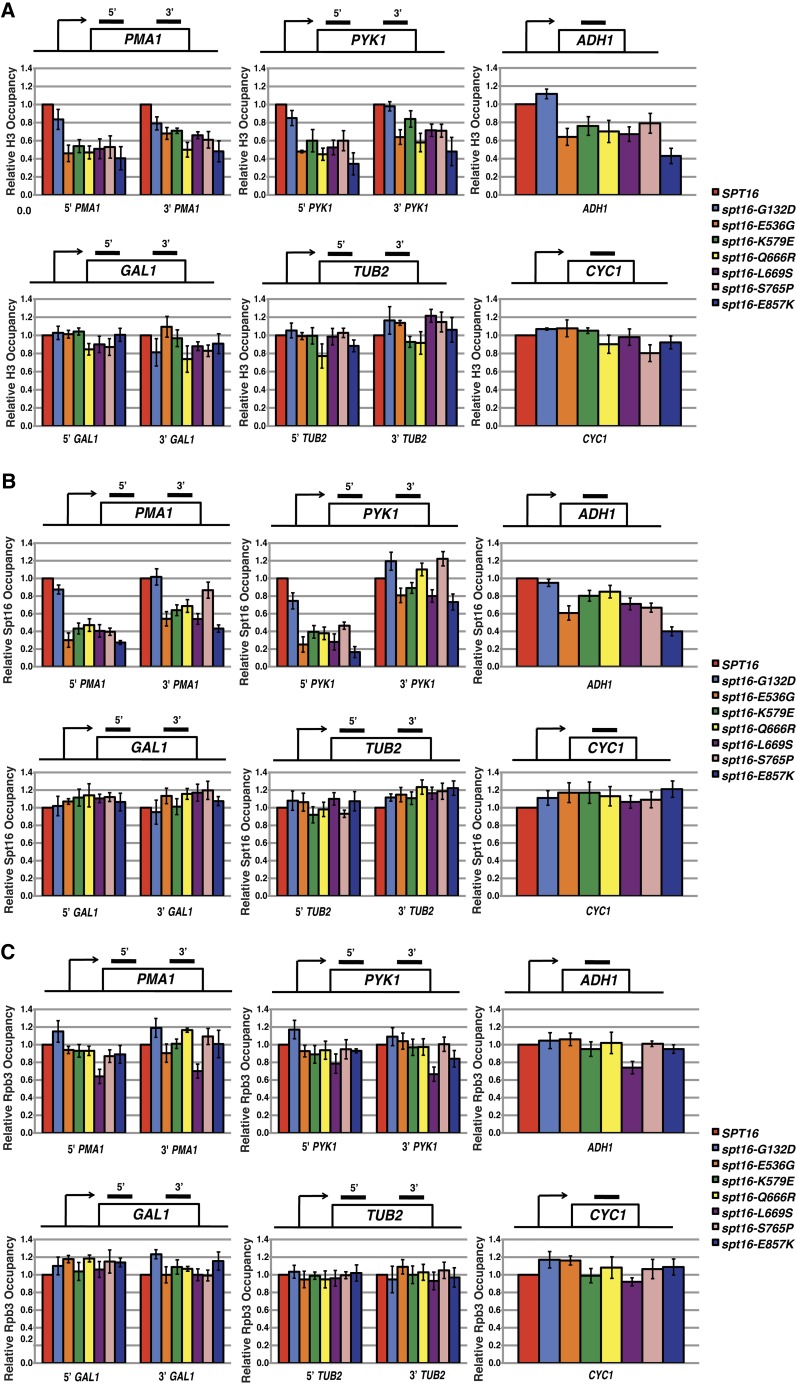
Relative occupancy of histone H3, Spt16, and Rpb3 across the coding regions of a subset of yeast genes. Histone H3 (A), Spt16 (B), and Rpb3 (C) was measured by ChIP within the coding region of three highly transcribed genes: *PMA1*, *PYK1*, and *ADH1* (top panels in A, B, and C) and over three lowly transcribed genes: *GAL1*, *TUB2*, and *CYC1* (bottom panels in A, B, and C) as described in Figure 6. The regions assayed by qPCR are marked with the black bars in the diagram provided for each gene. All values represent the mean ± SEM of three independent experiments.

To investigate whether the *spt16* mutants that reduce nucleosome occupancy across *SRG1* have a general defect in transcription-coupled nucleosome occupancy, we measured histone H3 occupancy across the coding sequences of a subset of yeast genes by ChIP (Figure 7A). At three highly transcribed genes, *PMA1* (100 mRNA/hr), *PYK1* (95 mRNA/hr), and *ADH1* (125 mRNA/hr) (Holstege *et al.* 1998), histone H3 levels were reduced in all of the mutants to a similar extent as we observed across *SRG1*. Conversely, histone H3 occupancy at three lowly transcribed genes, *GAL1* (repressed), *TUB2* (12 mRNA/hr), and *CYC1* (10 mRNA/hr) (Holstege *et al.* 1998), was unaffected in the mutants.

We next examined the occupancy of these mutant derivatives of Spt16 across the coding sequence of this subset of yeast genes (Figure 7B). Consistent with our results at *SRG1*, we found that at the highly transcribed genes, *PMA1*, *PYK1*, and *ADH1*, the binding of the mutant Spt16 proteins were generally reduced in accordance with the decrease in histone H3 levels across these regions. Interestingly, the decrease in the occupancy of these mutant Spt16 proteins was greater at the 5′ end of these genes compared with regions toward the 3′ end. For the most part, these changes in Spt16 binding occurred in the absence of any change in RNA Pol II binding to these regions (Figure 7C). Interestingly, a small but significant decrease in Rpb3 levels in the *spt16-L669S* mutant (*P* < 0.05) was detected at these highly transcribed genes, comparable to what we observed at *SRG1*. In contrast to what we observed at highly transcribed genes, occupancy of the mutant Spt16 proteins and Rpb3 at three lowly transcribed genes, *GAL1*, *TUB2*, and *CYC1*, were largely unaffected. Importantly, we show that when these mutants are grown in galactose-containing medium to induce high levels of *GAL1* expression, we detected reduced occupancy of both H3 and the mutant Spt16 proteins to the *GAL1* coding sequence similar to what we observed for *SRG1* and other highly transcribed genes (Figure S1). Thus, we have identified mutant *spt16* alleles that cause reduced occupancy of both the mutant version of Spt16 encoded by these alleles and histones specifically over highly transcribed regions of the genome. Taken together, our studies suggest that the integrity of the Spt16-D and Spt16-M domains is generally required to maintain nucleosome occupancy at highly transcribed genes, possibly by facilitating Spt16 recruitment to those genes.

## Discussion

Spt16 is an essential, highly conserved component of the FACT elongation complex with a dual role in transcription elongation—the disassembly of nucleosomes to allow the passage of RNA Pol II and their reassembly in the wake of RNA Pol II [reviewed in Duina (2011), Formosa (2011), Reinberg and Sims (2006), and Winkler and Luger (2011)]. In this work, we provide evidence indicating that the integrity of both the Spt16-D and Spt16-M domains are required to support the histone chaperone activities of Spt16 during transcription elongation. We utilized a previously characterized system in which this activity of Spt16 is required for *SRG1* transcription–dependent repression of the *S. cerevisiae SER3* gene (Hainer *et al.* 2011) to identify a largely novel class of mutations in *SPT16* that derepress *SER3*. Six mutations that most strongly derepress *SER3* contain single amino acid substitutions in either the Spt16-D or Spt16-M domain. For this subset of mutants, *SRG1* transcription–coupled nucleosome occupancy over the *SER3* promoter is reduced to degrees that generally correlate with *SER3* derepression. Moreover, we provide evidence that these mutations broadly disrupt transcription-coupled nucleosome occupancy at highly transcribed regions of the yeast genome. Finally, we show that while these mutant versions of Spt16 are expressed at wild-type levels, their association with highly transcribed genes is significantly reduced. These data suggest that the integrity of the Spt16-D and Spt16-M domains are required for transcription-coupled nucleosome occupancy, possibly by promoting or maintaining FACT association with transcribed regions of the genome.

With one exception (*spt16-E857K*), the *spt16* mutants that we identified in this work are distinct from those that have been previously identified by other genetic approaches (Formosa *et al.* 2002; Malone *et al.* 1991; Myers *et al.* 2011; O’Donnell *et al.* 2009; Stevens *et al.* 2011). Although most of this new class of *spt16* mutants confers an Spt^−^ phenotype similar to many previously characterized *spt16* mutants, additional phenotypic studies indicate that there are important functional differences between these mutants. First, these mutants do not confer lethality at elevated temperature as is common for many previously characterized *spt16* mutant alleles (Formosa *et al.* 2002; Myers *et al.* 2011; O’Donnell *et al.* 2009). This result suggests that the amino acid substitutions caused by these mutations are not likely to affect the general stability of the Spt16 protein. Furthermore, these results indicate that the ability of Spt16 to promote nucleosome assembly during transcription is not essential for viability. Second, these mutants do not confer a growth defect in the presence of hydroxyurea, a phenotype conferred by other *spt16* mutants (Formosa *et al.* 2002; Myers *et al.* 2011; O’Donnell *et al.* 2009) that is indicative of a defect in DNA replication and/or DNA repair (Hampsey 1997). Therefore, this new group of *spt16* mutants may define an activity for Spt16 that is specific to its role in transcription elongation rather than a histone chaperone activity that may be generally required for all of Spt16 functions. Third, these *spt16* mutants do not suppress a cold-sensitive growth defect conferred by a histone H3 L61W as has been recently described for a distinct set of *spt16* mutant alleles (Myers *et al.* 2011). This is somewhat surprising given that both groups of *spt16* mutants have amino acid substitutions within the Spt16-M domain. Moreover, one of the *spt16* mutants isolated as a suppressor of the cold sensitivity of the histone H3 L61W mutant contains a glutamine substitution of glutamic acid residue at position 847, the same residue that, when substituted for a lysine, confers strong *SER3* derepression and transcription-coupled nucleosome assembly defects. However, the lysine substitution did not suppress the cold sensitivity of the H3 L61W mutation. Taken together, these data show that we have identified a new class of *spt16* mutants that interferes with Spt16 activity specific to its role in transcription-coupled nucleosome assembly rather than its generally required functions in transcription, cell viability, and/or DNA replication/DNA repair.

During our phenotypic analyses, we found that most of the *spt16* mutants that were isolated based on their ability to derepress *SER3* also confer sensitivity to cyclohexamide, a phenotype that has not been previously described for *spt16* mutant alleles. Cyclohexamide is a potent inhibitor of eukaryotic protein synthesis that is normally toxic to yeast cells (Mccusker and Haber 1988). However, at low doses, a sensitivity to this drug has been shown to reveal mutations that reduce protein synthesis or impair cell-cycle progression (Hampsey 1997). Therefore, although the identification of this phenotype may be interesting, the interpretation of the data are unclear. We hypothesize that the subset of *spt16* mutants causing cyclohexamide sensitivity do so as a result of the misregulation of one or more genes encoding proteins that are essential for viability, regulate protein synthesis, or regulate intracellular levels of cyclohexamide.

Interestingly, the *spt16-E857K* allele, which we found to confer a dominant negative effect on *SER3* repression, was previously isolated as a dominant suppressor of the transcription defects of δ element insertions just 5′ of both the *LYS2* and *HIS4* genes (O’Donnell *et al.* 2009; Stevens *et al.* 2011). This is not surprising given the striking similarities between *SER3* repression by *SRG1* transcription and *LYS2* and *HIS4* repression by the δ element insertions (Clark-Adams and Winston 1987; Martens *et al.* 2004; Winston *et al.* 1984). Both *SRG1* and the δ element insertion promote transcription across the promoters of their adjacent genes, *SER3* and either *LYS2* or *HIS4*, respectively. Our finding that *SER3* derepression in the *spt16-E857K* mutant is the result of reduced *SRG1* transcription–dependent nucleosome assembly at the *SER3* promoter suggests that a similar transcription defect in nucleosome occupancy may play a role in alleviating repression of *LYS2* and *HIS4* caused by these δ element insertions. Interestingly, we found that whereas three of the five double mutants containing the *E857K* substitution also act in a dominant manner, two of these combinations, *spt16-I626T/E857K* and *spt16-T651A/H741Y/E857K*, do not. Moreover, we found that the level of *SER3* derepression in these two mutant alleles to be significantly lower to that caused by the *E857K* substitution alone (B. A. Charsar, unpublished data). Therefore, *I626T* and one or both of *T651A* and *H741Y* substitutions appear to suppress the negative effects of the *E857K* substitution.

Our analysis of the single amino acid substitutions in the Spt16-D and Spt16-M domains revealed a strong correlation between defective transcription-dependent nucleosome assembly and reduced association of these mutant versions of Spt16 at highly transcribed regions of the yeast genome. Several possible models could account for these observations. First, these mutant versions of Spt16 may interfere with the normal recruitment of FACT to transcribed DNA. In this model, the reduced recruitment of FACT would be the cause of the defect in transcription-coupled nucleosome assembly. Although several studies have determined that FACT physically associates with DNA that is being transcribed (Duina *et al.* 2007; Kim *et al.* 2004; Mason and Struhl 2003; Mayer *et al.* 2010), the molecular mechanism of this association is not known. Previous studies have implicated a number of factors that may facilitate Spt16 association with transcribed DNA, including the Chd1 chromatin-remodeling factor, the Paf1 elongation complex, RNA Pol II, and histone proteins (Adelman *et al.* 2006; Biswas *et al.* 2007; Formosa *et al.* 2001; Mason and Struhl 2003; Pruneski *et al.* 2011; Simic *et al.* 2003; Winkler *et al.* 2011). It is conceivable that the amino acid substitutions within the Spt16-D and Spt16-M domains that interfere with transcription-coupled nucleosome assembly do so by altering FACT interactions with one or more of these factors. Second, the reduction in Spt16 association with transcribed regions may be a consequence of the reduced nucleosome occupancy due to a defect in transcription-coupled nucleosome assembly. In this model, the amino acid substitutions in the Spt16-D and Spt16-M domains would not alter initial Spt16 recruitment to transcribed DNA or its ability to associate with nucleosomal DNA but, rather, interfere with its nucleosome remodeling activity that leads to disassembly and/or reassembly of nucleosomes during transcription. Additional molecular and biochemical experiments to investigate the affect of these mutants on FACT interactions with other proteins and the nucleosome remodeling activity of Spt16 will be necessary to distinguish between these models.

Although the possibility that the Spt16-D and Spt16-M domains may directly mediate protein-protein interactions or FACT nucleosome remodeling activity is intriguing, we cannot rule out a more indirect role for these domains. For example, it is possible that the three mutations in the Spt16-D domain may simply disrupt the Spt16-Pob3 interface (VanDemark *et al.* 2006, 2008). However, if this were the case, we would expect any changes in the Spt16-Pob3 dimer interface to be subtle, specifically affecting the activity of FACT in transcription-dependent nucleosome assembly rather than in a more general histone chaperone role. Large perturbations in the Spt6-Pob3 interaction would most likely lead to more broad defects in cell growth and DNA replication/repair, which were not detected in these mutants by our phenotypic assays.

In summary, we have identified a novel class of *spt16* mutants that specifically impairs transcription-coupled nucleosome occupancy across highly transcribed regions of the *S. cerevisiae* genome and results in reduced association of the mutant Spt16 proteins to these regions. These mutants are likely to be useful molecular tools to further elucidate the dynamic function of Spt16 in maintaining chromatin architecture during transcription.

## Supplementary Material

Supporting Information
