# Supplementary material for: Identification of Mutant Versions of the Spt16 Histone Chaperone That Are Defective for Transcription-Coupled Nucleosome Occupancy in Saccharomyces cerevisiae
Source: G3 (Bethesda). 2012 May 1;2(5):555–67. doi: 10.1534/g3.112.002451 (PMC3362939; doi:10.1534/g3.112.002451)
Supplement: Supporting Information [file supp_2_5_555__index.html]

Supporting Information 

# Identification of Mutant Versions of the Spt16 Histone Chaperone That Are Defective for Transcription-Coupled Nucleosome Occupancy in *Saccharomyces cerevisiae*

## Supporting Information for Hainer *et al*, 2012

**Files in this Data Supplement:**

- Supporting Information - Figure S1 and Table S1 (PDF, 169 KB)
- Figure S1 - Relative occupancy of histone H3, Spt16 and RNA Pol II in *spt16* mutants over *GAL1* (PDF, 148 KB)
- Table S1 - List of *Saccharomyces cerevisiae* strains used in this work (PDF, 77 KB)
