# Supplementary material for: Identification of Mutant Versions of the Spt16 Histone Chaperone That Are Defective for Transcription-Coupled Nucleosome Occupancy in Saccharomyces cerevisiae
Source: G3 (Bethesda). 2012 May 1;2(5):555–67. doi: 10.1534/g3.112.002451 (PMC3362939; doi:10.1534/g3.112.002451)
Supplement: Supporting Information [file supp_2.5.555_FigureS1.pdf]

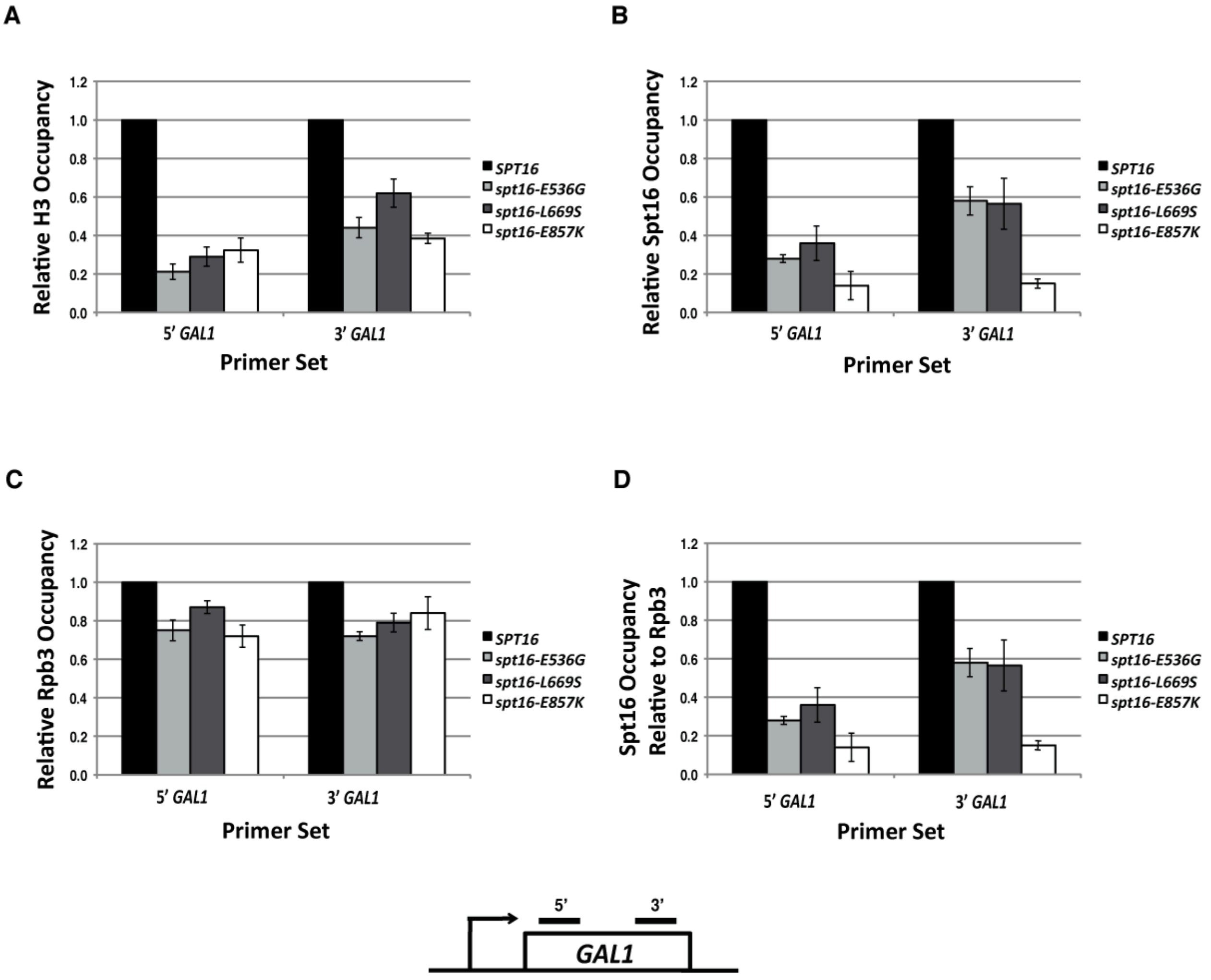

Figure S1 Relative occupancy of histone H3, Spt16 and RNA Pol II in *spt16* mutants over *GAL1*. Histone H3 (A), Spt16 (B), and Rpb3 (C) ChIP experiments were performed on chromatin prepared from *spt16Δ* strains containing either plasmid-borne copies of either wild-type *SPT16* or the indicated *spt16* mutant alleles, which were grown in YPRaff at 30°C and shifted to YPGal for 1 hr. The amount of immunoprecipitated DNA at 5' and 3' locations within the *GAL1* open reading frame (indicated by black bars in the diagram of *GAL1* below the graphs) was determined by qPCR as a fraction of the input material and normalized to a control region in chromosome V. Each bar represents the mean +/- SEM of three independent experiments using strains derived from YJ1091 or YJ1092. Occupancy of these factors in the strains expressing wild-type Spt16 was arbitrarily set to 1 at each *GAL1* location. D) Occupancy of Spt16 over *GAL1* was recalculated relative to Rpb3 occupancy.
