# Supplementary material for: Identification of Mutant Versions of the Spt16 Histone Chaperone That Are Defective for Transcription-Coupled Nucleosome Occupancy in Saccharomyces cerevisiae
Source: G3 (Bethesda). 2012 May 1;2(5):555–67. doi: 10.1534/g3.112.002451 (PMC3362939; doi:10.1534/g3.112.002451)
Supplement: Supporting Information [file supp_2.5.555_TableS1.pdf]

Table S1 List of *Saccharomyces cerevisiae* strains used in this work

Strain	Genotype	Reference or Source
YJ920	*MATα ura3Δ0 leu2Δ0 his3Δ200 lyp1Δ::SER3pr-URA3*	Hainer and Martens, 2011
YJ1089	*MAT***a** *ura3Δ0 leu2Δ0 his3Δ200 lyp1Δ::SER3pr-HIS3 spt16Δ::KanMX <pSPT16-URA3>*	This study
YJ1090	*MAT***a** *ura3Δ0 leu2Δ0 his3Δ200 lyp1Δ::SER3pr-HIS3*	This study
YJ1091	*MATα ura3Δ0 leu2Δ0 lys2-128δ trp1Δ63 spt16Δ::KanMX <pSPT16-URA3>*	This study
YJ1092	*MAT***a** *ura3Δ0 leu2Δ0 his3Δ200 spt16Δ::KanMX KanMX-GAL1pr-FLO8-HIS3 <pSPT16-URA3>*	This study
YADP50	*MATα his3Δ200 leu2Δ1 ura3-52 lys2-128δ (hht1-hhf1)Δ::HIS3 hht2-11 spt16Δ::KanMX pSPT16*	Myers et al, 2011
